# False belief understanding and “cool” inhibitory control in 3-and 4-years-old Italian children

**DOI:** 10.3389/fpsyg.2015.00872

**Published:** 2015-06-29

**Authors:** Francesca Bellagamba, Elsa Addessi, Valentina Focaroli, Giulia Pecora, Valentina Maggiorelli, Beatrice Pace, Fabio Paglieri

**Affiliations:** ^1^Dipartimento di Psicologia Dinamica e Clinica, Sapienza Università di RomaRome, Italy; ^2^Istituto di Scienze e Tecnologie della Cognizione, Consiglio Nazionale delle RicercheRoma, Italy; ^3^Università Campus Bio-MedicoRoma, Italy; ^4^Dipartimento di Psicologia dei Processi di Sviluppo e Socializzazione, Sapienza Università di RomaRome, Italy

**Keywords:** false belief, inhibitory control, Italian preschoolers, conflict task, delay task, delay choice task

## Abstract

During preschool years, major developments occur in both executive function and theory of mind (ToM), and several studies have demonstrated a correlation between these processes. Research on the development of inhibitory control (IC) has distinguished between more cognitive, “cool” aspects of self-control, measured by conflict tasks, that require inhibiting an habitual response to generate an arbitrary one, and “hot,” affective aspects, such as affective decision making, measured by delay tasks, that require inhibition of a prepotent response. The aim of this study was to investigate the relations between 3- and 4-year-olds’ performance on a task measuring false belief understanding, the most widely used index of ToM in preschoolers, and three tasks measuring cognitive versus affective aspects of IC. To this end, we tested 101 Italian preschool children in four tasks: (a) the Unexpected Content False Belief task, (b) the Conflict task (a simplified version of the Day–Night Stroop task), (c) the Delay task, and (d) the Delay Choice task. Children’s receptive vocabulary was assessed by the Peabody Picture Vocabulary test. Children’s performance in the False Belief task was significantly related only to performance in the Conflict task, controlling for vocabulary and age. Importantly, children’s performance in the Conflict task did not significantly correlate with their performance in the Delay task or in the Delay Choice task, suggesting that these tasks measure different components of IC. The dissociation between the Conflict and the Delay tasks may indicate that monitoring and regulating a cool process (as flexible categorization) may involve different abilities than monitoring and regulating a hot process (not touching an available and highly attractive stimulus or choosing between a smaller immediate option and a larger delayed one). Moreover, our findings support the view that “cool” aspects of IC and ToM are interrelated, extending to an Italian sample of children previous findings on an association between self-control and ToM.

## Introduction

Over the past 20 years there have been significant improvements in research on two milestones of cognitive development, theory of mind (ToM) and executive functioning (EF). In both these domains of cognition, major developments occur between the ages of 3 and 5 years.

Theory of mind is the ability to attribute mental states, such as emotions, beliefs, and intentions to oneself and to other people. Mental state understanding helps the child to make behavioral predictions about how people will act (Perner and Lang, 1999). An important transition in the development of a ToM, emerging around the age of 4 years, is the explicit understanding that a person can be mistaken about the world, that is, the comprehension of false belief and the distinction between appearance and reality (Perner et al., 1987). Before this age, children have difficulties in understanding that a false belief can cause one to search for an object in the wrong place, and children tend not to manipulate other people’s behavior by lying or deceiving (Sodian et al., 1991).

Prototypical tests for ToM, including the Deceptive Container task and the Appearance- Reality task, measure the representational nature of mental states, and are failed by most preschoolers at age 3 but are grasped by age 5. The most frequently used measure for assessing ToM at around 4 years of age is the ‘False-Belief task.’ The standard version requires the unexpected transfer of a wanted object, so that the protagonist has a false belief about the location of the object, and children are asked to predict where the protagonist will look for the object (Perner and Lang, 1999). At 3 years of age almost all children answer wrongly with the actual location of the object, whereas most children of 4 years and older answer correctly. False belief performance shows a similar developmental pattern across various countries and task manipulations: preschoolers progress from below-chance performance to above-chance performance, suggesting that understanding of belief and mind exhibits conceptual change in the preschool years (Wellman et al., 2001, 2006).

Some studies have suggested that having one or more siblings to interact with at home promotes ToM understanding (Perner et al., 1994; Jenkins and Astington, 1996). Children with siblings have access to other children’s mind via arguments, reciprocal engagement in pretend play and child-oriented conversation (McAlister and Peterson, 2013). Moreover, many studies have shown that high-functioning children with autism spectrum disorders exhibit deficits in ToM understanding as measured by False Belief tasks. These deficits do not emerge in control groups of subjects with Down’s syndrome, general retardation or specific language delays (Sodian and Frith, 1992; Baron-Cohen, 1995).

Executive functioning (EF) refers to higher-order self-regulatory cognitive processes that enable a person to engage in flexible goal-directed behaviors, including the control of attention, and motor responses, resistance to interference and delay of gratification (Carlson et al., 2004a). EF has been frequently associated with the prefrontal cortex, which is one of the slowest developing brain areas; also, EF is generally regarded as non-social and domain general (Hughes and Ensor, 2007). There is a growing evidence that executive function is not a unitary construct, but rather involves a series of distinct processes under the control of the frontal lobe, including working memory, IC, and task switching (Garon et al., 2008; Duckworth and Kern, 2011; Miyake and Friedman, 2012). Important developments in typically developing children in IC occur in the first 6 years of life. The first signs of inhibition (such as the ability to ignore distraction and stay focused, or to resist making a habitual response to produce a new and more adaptive one), are evident by 12 months of age, when infants succeed in the A-not-B and object retrieval tasks (Diamond, 2006). In the preschool period, children make important improvements in self-control over actions, thoughts and emotions (Carlson, 2005; Lewis and Carpendale, 2009). Three-years-old children have difficulty in waiting for a reward, in staying on-task in the face of tempting distractions, and in learning a reverse-reward contingency task (in which they should point to a small amount of candies in order to receive a larger amount). In contrast, 4-year-olds are able to exert more self-control, and in the reverse-reward contingency task they point to the undesired option in order to get the other one (Carlson et al., 2005).

Although EF is considered a domain-general construct, a distinction has been made between the relatively hot affective aspects of EF and more purely cognitive, cool aspects (Metcalfe and Mischel, 1999; Zelazo and Muller, 2002). Whereas cool EF is more likely to be involved in relatively abstract, decontextualized problems, hot EF is required when the regulation of affect and motivation is solicited by the task (Zelazo et al., 2005). Metcalfe and Mischel (1999) formulated the hypothesis of a brain network model in which self-control occurs through the interplay between a bottom–up, affective ‘go’ system, labeled the “hot system,” and a top–down, cognitive system, labeled the “cool system.” The hot system develops earlier and is under stimulus control. The cool system develops later and is under self-control. Involvement of the hot system may be related to an over-focusing on visible reward and lower self-control. Garon et al. (2012) used this model to interpret the results of a recent study on the development of future-oriented self-control. The study explored factors underlying 2-, 3-, and 4-years-old preschoolers’ capacity to make future-oriented choices using a delay-of-gratification choice task. When choosing between two reward, with the larger being delayed, children have to consider two variables – the quantity of the reward, which implicates the bottom–up system, and the temporality of the reward, which implicates the top–down system. Their findings indicated that children made more choices to delay gratification as the quantity of the reward increased. Looking at age-related differences, Garon et al. (2012) argue that, while 2-year-olds focused on quantity and 3-year-olds showed a mixed pattern, 4-year-olds were able to consider both time and quantity together in making their choices.

Two different classes of tasks have been used to measure IC in the preschool period (Carlson and Moses, 2001). The first class (Delay tasks) evaluates affective (“hot”) aspects of IC and includes measures of children’s ability to delay, control, or suppress an impulsive response. An example of this class of tasks is the Gift delay task (Kochanska et al., 2000), in which an experimenter tells the child not to have a look while the experimenter noisily wraps a present. Children’s waiting ability on this and other ‘delay’ tasks gets better across the preschool years. Another classical delay measure is the Delay of gratification task (Mischel et al., 1989). In this task, 4-years-old children waited longer to receive a larger reward (two marshmallows rather than one) when they were encouraged to cognitively transform the reward (for instance, by imagining the marshmallows as fluffy clouds) rather than when they were told to focus on the arousing qualities of the reward (i.e., its taste). A distinctive feature of Delay tasks is that the child must maintain a course of action in the face of continual competition from an available, tempting alternative. In fact, after the initial choice of delaying gratification, the immediate reward remains available throughout the delay; thus, the decision to wait for the preferred reward needs to be sustained during the entire delay, since the child can reverse the initial choice at any time by picking the smaller or less preferred item. This is different from what happens in the Delay Choice task, which is considered a further measure of “hot” IC. In this task, the subject faces a choice between a smaller immediate option and a larger delayed option and, once the choice is made, there is no possibility to modify it (Addessi et al., 2014). The second class (Conflict tasks) evaluates more cognitive (“cool”) aspects of IC and requires children to inhibit an habitual response to generate an arbitrary one. An example of conflict task is the Day–Night Stroop task developed by Gerstadt et al. (1994). This task requires children to say ‘day’ when a black card depicting the moon and the stars is shown and ‘night’ when a white card depicting a yellow sun is shown; thus, the expected response has to be suppressed. As with delay tasks, children’s performance on this and similar conflict tasks improves gradually during the preschool years.

The link between ToM and EF was first noted in the context of research on individuals with autism. Ozonoff et al. (1991) found that high-functioning children with autism were impaired both on measures of ToM and on tasks assessing EF, and suggested that the maturation of the same brain structures that underlie ToM and EF may be the cause of the observed correlations. Later studies strongly supported a general link between ToM and EF in typically developing preschoolers, and highlighted a special connection between ToM and IC (Carlson and Moses, 2001; Perner et al., 2002; Carlson et al., 2004a; Sabbagh et al., 2006; Hughes and Ensor, 2007; Henning et al., 2011; McAlister and Peterson, 2013). The correlations between individual differences in EF performances and ToM tasks in these studies are notable and remain even when factors such as age and verbal ability are controlled. A few studies suggested that the link between ToM and EF may be present even earlier than the preschool years, when measuring these emerging abilities may be challenging (Carlson et al., 2004a; Hughes and Ensor, 2005; Bellagamba et al., 2014; Poulin-Dubois and Yott, 2014). Despite the large number of studies that addressed the relationship between ToM and EF, only a few studies included both delay and conflict components of IC in their measures. Carlson and Moses (2001) examined the relation between individual differences in IC and ToM performance in preschool children of 3- and 4-years of age using a variety of tasks. The ToM battery incorporated measures of false belief, deceptive pointing and appearance-reality. The EF battery (10 measures) included conflict tasks and delay tasks. A multiple regression analysis revealed that the Conflict scale was a highly significant predictor of ToM, holding the control variables (age, gender, and verbal ability) and the Delay scale constant. The Delay scale, however, did not contribute uniquely to variance in ToM over and above the control variables and the Conflict scale. The authors noted that the abilities assessed by Conflict tasks may have been more central to ToM reasoning than those assessed by Delay tasks. In a follow-up study, Carlson et al. (2002) again found a different pattern for conflict and delay measures. The conflict tasks correlated with ToM controlling for age and intelligence. In contrast, the correlation between the Delay tasks and ToM was not significant. The authors hypothesized that the Conflict tasks impose loads on both working memory and inhibitory capacity, whereas the Delay tasks impose a substantial inhibitory load but only minimal working memory demands (Carlson and Moses, 2001; Carlson et al., 2002).

Hala et al. (2003) examined the relation between false belief understanding and executive function including a battery of both conflict and delay tasks. They found no relationship between the gift delay task and ToM, in contrast to a strong association between conflict and ToM scores. Like Carlson and Moses (2001), also Hala et al. (2003) suggest that the difference between conflict and delay tasks is principally in the working memory: conflict tasks demand that children keep in mind the pertinent rules as well as inhibit an impulsive response. Kain and Perner (2005), however, noted that the claim that the delay task poses lower memory demands than the conflict task is not very convincing because in delay tasks children have to keep reminding themselves for some time that they were instructed not to touch a forbidden object. Kain and Perner (2005) suggested that emotional and reward factors could provide another reason why the delay task bears a lower and less robust correlation with ToM tasks than does the conflict task. These authors noted that the delay tasks activate emotional and reward processing, which are known from other tasks (e.g., gambling task; Bechara et al., 1998) to be associated to the orbitofrontal cortex, whereas conflict tasks activate the dorsolateral prefrontal cortex and the anterior cingulated cortex, which are also involved in ToM.

Bellagamba et al. (2014) investigated the concurrent relations between 18- and 24-month-olds’ performance on two tasks measuring inhibitory control (IC; a Conflict and a Delay task) and internal state language abilities in 61 Italian speaking children and found that the ability to refer to mental states through language was significantly and specifically related only to performance on the conflict measure of IC, even when vocabulary size was controlled for.

Carlson et al. (2004b) examined the relative contribution of two aspects of executive function – IC and planning ability – to ToM in 3- and 4-year-olds. Children were given two standard ToM measures (Appearance–Reality and False Belief), three IC tasks (Bear/Dragon, Whisper, and Gift Delay), three planning tasks (Tower of Hanoi, Truck Loading, and Kitten Delivery), and a receptive vocabulary test. Multiple regression analyses indicated that only the two conflict inhibition tasks (Bear/Dragon and Whisper) were significantly related to ToM after accounting for age, receptive vocabulary, and planning, while the Gift Delay task was not. Finally, in a very recent study, Carlson et al. (2015) found similar results, showing that children’s better performance on a conflict task (Bear/Dragon) predicted higher scores in ToM tasks that presented both low and high levels of executive demands (Think-Know and Sources of Knowledge were low demanding, in addition to False Belief and Appearance-Reality, which were high demanding). In contrast, once again, the Gift Delay task was not related to either of the two kinds of ToM tasks.

A recent meta-analytic review of 102 studies reported a moderate to strong association between EF and false belief understanding in early childhood (Devine and Hughes, 2014), indicating that among typically developing 3- to 6-years olds there is a genuine association between individual differences in EF and false belief understanding. Also, the correlation between EF and false belief understanding was similar in magnitude from ages 3 to 6, and this consistency is remarkable as this developmental period is associated with rapid gains in both domains. Moreover, Devine and Hughes (2014) noted that false belief understanding is more strongly associated with conflict inhibition than with performance in the Gift Delay task and Sticker Delay task, but that only a very few studies evaluating the relationship between ToM and EF included also measures of delay of gratification in their analyses. Devine and Hughes (2014) also underlined that the Delay task measure cannot be considered equivalent to the Delay of Gratification task, since only the second task presents the child with a choice between a smaller reward now and a larger reward later.

On the basis of the above findings, the main goal of the present study was to examine the relationships between explicit false belief understanding, a delay measure of IC and a conflict measure of IC in a group of typically developing Italian children, controlling for age and receptive vocabulary. To our knowledge, this is the first study analyzing the relation between false belief understanding and IC in preschoolers belonging to this population [although a study by Valle et al. (2015) has recently addressed this issue in adolescents and early adults]. Our understanding of how children develop in ToM and EF is largely based on Anglo-American, French- and German-speaking children, but there is an increasing focus on cultural differences in the development of children’s understanding of mind (Lillard, 1998). Culture plays an important role in shaping how parents think and act out their parental role (Bornstein, 1991), which in turn interacts with universal pathways of infant development. Hsu and Lavelli (2005) found both cross-cultural similarities and distinctive differences in social/affective aspects of feeding between Italian and American mothers. In their interactions with their infants, Italian mothers tend to promote the expression of positive affect and social relatedness with others, while American mothers tend to encourage independence and self-reliance. According to Lecce and Hughes (2010), cultural differences in the expression of emotions and in the focus on self-control could contribute to the differences in ToM performances in British and Italian children. Parental education, different styles in maternal use of mental state terms, and differences in the onset of formal schooling – which begins at age 5 in Britain, but at age 6 in Italy – are considered factors that may also contribute to the advantage observed in their study by British children on false belief understanding, compared to a matched sample of Italian children. Also, findings on the relation between ToM and EF from cross-cultural studies appear mixed. Sabbagh et al. (2006) suggested that children growing in two very different cultures, China and the United States, showed considerable cross-cultural synchrony in the association between ToM and EF tasks, suggesting that this relation may be universal and not changed by cultural differences. A later study, involving children from three oriental cultures, instead suggested that the patterns of executive skills and their correlates with standard false belief measures are very different from those found in Western cultures (Lewis et al., 2009). Oriental children tend to outperform Western children on executive function tasks, whereas they do not exhibit these advanced levels of performance in false belief tests. The above study also reports a lack of association between false belief understanding and EF composite measures for Korean, Japanese, and Chinese children. Therefore, as noted by Devine and Hughes (2014), a systematic comparison of the impact of cultural differences is important to understand whether the relation between EF and ToM does vary in strength across different cultures.

Given that previous studies have reported an association between ToM tasks and conflict tasks in children between 3 and 6 years of age (Carlson and Moses, 2001; Perner et al., 2002; Carlson et al., 2004a; Hughes and Ensor, 2007; Henning et al., 2011), we hypothesized that there would be a stronger relation between ToM and the conflict measure of IC rather than between ToM and the delay measure of IC. In line with the proposal of Kain and Perner (2005), we expected false belief understanding to be more strongly related to a task requiring the child to overcome a dominant response and start a conflicting one, than to a task measuring the capacity to delay a response toward an highly attractive stimulus.

## Materials and Methods

Data were collected during a study on children’s self-control ability, focusing on how symbolic representations of the reward affected performance in a Delay Choice Task (Addessi et al., 2014), and whether displacement activities improved children’s performance in the Delay Task (Pecora et al., 2014). Data for the Delay Choice Task, Conflict Task, and Delay Task were partly analyzed in the aforementioned studies, whereas data for the False Belief task are completely original.

### Participants

Participants were 101 Italian preschool children, 51 3-year-olds (mean age = 36.13, range = 35.07–37.0; 25 boys and 26 girls) and 50 4-year-olds (mean age = 48.11, range = 47.02–49.0; 27 boys and 23 girls). Children were sourced from kindergarten and were all healthy. They came from middle-class Italian families (as determined by parental educational level) living in Rome. The children’s parents signed an informed consent form outlining the aim of the study. The study complied with the ethical guidelines of the Italian Association of Psychology (AIP) and were approved by the Ethics Committee of ISTC-CNR.

### Procedure

Tasks were administered to the children in a quiet room by two qualified experimenters, who alternated their role as experimenter and assistant across sessions. The experimenter administered the tasks and the assistant quietly recorded children’s performance on the protocol sheet. The entire session was also video-recorded. Each child was given five tasks in one single session and in a fixed order: (a) the Delay choice task, (b) the Peabody Picture Vocabulary Test (PPVT, Dunn and Dunn, 1981), (c) the Unexpected Content False Belief Task (Perner et al., 1987; Gopnik and Astington, 1988), (d) the Conflict Task, a simplified version of the Day–Night Stroop Task (adapted from Gerstadt et al., 1994), and (e) the Delay Task (adapted from Vaughn et al., 1986 and Kochanska et al., 2000). Depending on the parents’ preference, the experiment was administered either in a separate room of the kindergarten that the child attended (65% of 3-year-olds, and 78% of 4-year-olds) or at home. Each testing session lasted around 40 min. At the end of the experiment, each child was given a small gift and the parents were given a DVD of the recorded experiment to thank them. Data collection was carried out between November 2009 and May 2011.

### Measures

#### Delay Choice Task

Children were presented with choices between a small option and a large option in three experimental conditions (Food Delay, Low-Symbolic Token Delay, and High-Symbolic Token Delay), in which the smaller option was immediately available, whereas the larger option was delayed by 80 s. Children were also tested in two control conditions (Food Control and High-Symbolic Token Control), in which both options were immediately available. We employed a between-subject design, counterbalancing gender and age. Each subject participated in a single session of six trials, including two familiarization trials (forced choices, with only one option available, presented at the beginning of the session), and four experimental trials (binary choices). In all conditions, the dependent variable was the proportion of choices of the larger delayed option.

In the Food conditions, children chose between visible food amounts, whereas in both Token conditions children were presented with two cards depicting, respectively, two dots and six dots (Low-Symbolic Token condition) or a mouse and an elephant (High-Symbolic Token condition). After choosing one of the two cards, the subject could exchange it with the experimenter for obtaining the corresponding food amount. The food type was previously agreed upon with the parents on the basis of children’s preferences and/or diet restrictions. For further details on the methodology, please see Addessi et al. (2014).

#### Peabody Picture Vocabulary Test

We used the Italian version of the PPVT-R (Dunn and Dunn, 1981, adapted by Stella et al., 2000), a measure of receptive vocabulary. For each item out of a series of 175 items, the child was asked to select from a set of four pictures the one best illustrating the meaning of an orally presented word. Testing continued until the child erred on eight consecutive items.

#### False Belief Task

We used the Unexpected Content False Belief Task (Perner et al., 1987; Gopnik and Astington, 1988). The children were shown a container of a desirable and highly familiar candy (a Smarties box) and asked to state what they thought was in the container (Control question). The experimenter then opened the container and showed that the box actually contained something unexpected (a pencil). The pencil was then put back into the box and the box closed again. The subjects were then asked the first experimental question about their own former false belief: “When you first saw this box, before we opened it, what did you think was inside?” Then they were asked the second experimental question, about someone else, Mary, who had never looked inside the box: “What will Mary think is in here?” The children were assigned either a 0 (fail) or a 1 (pass) for the three different scores: (1) Self attribution, (2) Other attribution, and (3) Global score (passing both self- and other attribution). Intercoder reliability, calculated on 20% of the sample, was 100% (index of concordance).

#### The Conflict Task

This task was a simplified version of the Day–Night Stroop Task developed by Gerstadt et al. (1994) and was used to assess the children’s capacity to inhibit a prepotent response in order to give a conflicting one. Both Gerstadt et al. (1994) and Waston and Bell (2013) indicate that the Day–Night Stroop task is too difficult for 3-years-old children. We thus employed a simpler version of the task using one set of five red and five blue cards. The experimenter first verified that the child correctly named the color of a red and of a blue card and then said: “Now we are playing a game. In this game, when I show you a red card you have to say ‘blue’ and when I show you a blue card you have to say ‘red,’ okay?” Two training trials followed in which the children were shown one of each type of card. If the subject hesitated, the experimenter prompted the subject by saying: “What do you say for this one?” If the subject responded correctly to the red card, the experimenter praised the child and proceeded to a training trial with the blue card. If the subject responded incorrectly or did not respond at all on either of these trials, the experimenter immediately reminded the subject of the rule. On the two training trials, the experimenter gave feedback and repeated the question as needed (up to three times). The experimenter then proceeded to the eight test trials without feedback in a fixed random order. The experimenter reminded the subject of the rule of the game after the first four test trials. The dependent variable was the total number of correct responses in the test trials. Intercoder reliability, calculated on 20% of the sample, was 100% (index of concordance).

#### The Delay Task

This task was adapted from Vaughn et al. (1986) and Kochanska et al. (2000), and was used to assess the children’s capacity to delay responding to an attractive stimulus. The experimenter showed a musical box to the child, with a carillon inside that turned on once the box was opened, and explained that the experimenter would have to leave the room for a short time. The child was then instructed not to touch the box in the experimenter’s absence. The experimenter and the assistant left the room and waited for 3 min, but the child could interrupt the trial at any time before the 3 min elapsed. During the delay period, the child received four yellow toy ducks to play with.

Five different scores were derived from this task: (1) *Latency to touch the box*: total time from task onset until the subject touched the box, (2) *Latency to open the box*: total time from task onset until the subject opened the box, (3) *Frequency of touching the box*: number of times the subject touched the box during the 3 min (or until the end of the trial if the child interrupted the trial earlier), (4) *Frequency of opening the box*: number of times the subject opened the box during the 3 min (or until the end of the trial if the child interrupted the trial earlier), and (5) *Time to interruption*: total time from task onset until the subject interrupted the trial or the 3 min elapsed. All measures were scored from videotapes. Intercoder reliability was calculated on 20% of the sample (*Latency to touch the box*: Spearman *r*_s_ = 1.0, *N* = 19; *Latency to open the box*: Spearman *r*_s_ = 0.99, *p* < 0.0001, *N* = 19; *Frequency of touching the box*: index of concordance: 91%; *Frequency of opening the box*: index of concordance: 95%). Intercoder reliability was not calculated for *Time to interruption* since most of the children (72%) waited until the end of the task.

## Results

Descriptive Statistics for All Tasks are Presented in **Table 1**.

**Table 1:** Mean scores, SD, and ranges for all tasks, divided by age.

Task	Measure	3-years-old			4-years-old		
		*M*	SD	Range	*M*	SD	Range
Peabody Picture Vocabulary Test (PPVT)	Score	77.6	7.19	67–98	90.8	10.5	68–119
False Belief Test	Self-attribution (score)	0.42	0.50	0–1	0.68	0.47	0–1
	Other attribution (score)	0.19	0.39	0–1	0.38	0.49	0–1
	Global score	0.15	0.36	0–1	0.30	0.46	0–1
Conflict task	Correct responses (proportions)	0.45	0.39	0–1	0.78	0.31	0–1
Delay task	Latency to touch (s)	100.0	58.51	1–180	113.7	69.7	2–180
	Latency to open (s)	139.8	54.7	3–180	153.8	46.9	31–180
	Frequency to touch (proportions)	0.007	0.009	0–0.3	0.012	0.020	0–0.080
	Frequency to open (proportions)	0.003	0.007	0–0.3	0.007	0.002	0–0.010
	Time to interruption (seconds)	158	39.06	49.21–180	159.5	41.19	47–180
Delay Choice Task – Food Delay	Choice of the larger option (proportions)	0.70	0.28	0.25–1	0.65	0.27	0.25–1
Delay Choice Task – Low-Symbolic Token Delay	Choice of the larger option (proportions)	0.57	0.29	0.25–1	0.67	0.31	0–1
Delay Choice Task High-Symbolic Token Delay	Choice of the larger option (proportions)	0.50	0.24	0–0.75	0.48	0.07	0.25–0.50

### Delay Choice Task

All 101 children participated in this task (51 were 3-year-olds and 50 were 4-year-olds), 20 in each condition, with the exception of the High-Symbolic Token Delay condition in which there were 21 children. For analysis purposes, data were transformed by calculating the arcsin squareroot of proportions of choices of the larger option. As reported in Addessi et al. (2014), a factorial ANOVA with gender and age as between-subject factors revealed a main effect of condition, but no significant effect of gender and age, nor any significant interaction. Here we do not discuss the effect of condition since this was the focus of Addessi et al. (2014).

### Peabody Picture Vocabulary Test

All the children participated in this task, but data for one 4-years-old are not available because of video camera failure. We performed a factorial ANOVA with the children’s standardized score on the PPVT as the dependent variable and with gender and age as independent variables. There was a main effect of age: *F*_1,96_ = 53.9, *p* < 0.001, = 0.36, with 4-year-olds (*M* = 90.8, SD = 10.5) performing better than 3-year-olds (*M* = 77.6, SD = 7.19). Gender did not significantly affect performance (*F*_1,96_ = 1.01, *p* = 0.32), nor was there any significant interaction between gender and age (*F*_1,96_ = 1.21, *p* = 0.27).

### False Belief Task

Ninety-eight children participated in this task (48 were 3-year-olds and 50 were 4-year-olds). Three children did not participate because they did not answer the experimenter’s questions (two 3-year-olds) or did not have any experience with the candies (Smarties) used in the task (one 3-years-old). All children passed the control question, 42% of the 3-year-olds and 68% of the 4-year-olds passed the first experimental question, 19% of the 3-year-olds and 38% of the 4-year-olds passed the second experimental question, 15% of the 3-year-olds and 30% of the 4-year-olds passed both first and second experimental questions.

For each measure (First experimental question Self-attribution, Second experimental question Other attribution, and Global score), we performed a logistic regression with the children’s score as dependent variable, and with gender and age as independent variables. For both experimental questions, age significantly predicted performance in the False Belief task (First experimental question Self-attribution: *z* = 2.59, *p* = 0.010, 4-year-olds: *M* = 0.68, SD = 0.47, 3-year-olds: *M* = 0.42, SD = 0.50; Second experimental question Other attribution: *z* = 2.06, *p* = 0.040, 4-year-olds: *M* = 0.38, SD = 0.49, 3-year-olds: *M* = 0.19, SD = 0.39). For the Global score (passing both first and second experimental questions), there was a non-significant trend of 4-year-olds (*M* = 0.30, SD = 0.46) to perform better than 3-year-olds (*M* = 0.15, SD = 0.36; *z* = 1.79, *p* = 0.074). For all measures, gender did not significantly predict performance (First experimental question-Self attribution: *z* = 0.15, *p* = 0.88; Second experimental question Other attribution: *z* = -0.57, *p* = 0.57; Global score: *z* = -0.20, *p* = 0.84), nor was there any significant interaction between gender and age (First experimental question Self-attribution: *z* = -0.16, *p* = 0.87; Second experimental question Other attribution: *z* = -0.96, *p* = 0.33; Global score: *z* = -1.36, *p* = 0.17).

### Conflict Task

Eighty-one children participated in this task (36 were 3-year-olds and 45 were 4-year-olds). Twenty children did not participate because they failed to recognize the colors (seven 3-year-olds and two 4-year-olds), did not answer the experimenter’s questions (six 3-year-olds) or were tested when this task had not yet been introduced in the present study. Since one child had some invalid trials, we converted the number of correct responses into proportions. For analysis purposes, data were transformed by calculating the arcsin squareroot of proportions. As reported in Addessi et al. (2014), a factorial ANOVA with gender and age as between-subject factors revealed a main effect of age, with 4-years-old children (*M* = 0.78, SD = 0.31) performing better than 3-years-old children (*M* = 0.45, SE = 0.39). Gender did not significantly affect performance, nor was there any significant interaction between gender and age. The children’s proportion of correct responses in this task did not significantly correlate with their total latency to respond (combined for the eight trials), controlling for chronological age and receptive vocabulary (*r*_p_ = 0.19, *p* = 0.091, *N* = 80).

### Delay Task

Ninety-eight children participated in this task (48 were 3-year-olds and 50 were 4-year-olds). Three children did not participate because their caregiver was present during the task (two 3-year-olds) or because of video camera failure (one 3-years-old). Since the children could interrupt the trial at any time before the 3 min elapsed, we converted frequencies of touching and opening the box into proportions. Data were transformed by calculating the logarithm of latencies and the arcsin squareroot of proportions. We performed a MANOVA with, as dependent variable, the five measures scored during the Delay Task (*Latency to touch the box*, *Latency to open the box*, *Frequency of touching the box*, *Frequency of opening the box*, and *Time to interruption*). Gender and age did not significantly affect performance (Gender: λ = 0.97, *F*_5,90_ = 0.59, *p* = 0.71; Age: λ = 0.92, *F*_5,90_ = 1.53, *p* = 0.19), nor was there any significant interaction between gender and age (λ= 0.94, *F*_5,90_ = 1.09, *p* = 0.37).

### Relations between Tasks

First of all, we examined whether there was a correlation between our IC measures and, if so, whether this relation would remain after we controlled for age and receptive vocabulary. As for the Delay Choice Task, we analyzed only data for the experimental conditions (Food Delay, Low-Symbolic Token Delay, and High-Symbolic Token Delay).

The next series of analyses was aimed at specifying the relative contribution of cool IC (as measured by the Conflict Task) and hot IC (as measured by the Delay Choice Task and by the Delay Task) to ToM. As shown in **Table 2**, the correlations between the Other attribution and Global score measures of the False Belief Task, respectively, and performance in the Conflict task were significant, whereas the correlations between ToM and (i) performance in the Delay Choice Task and (ii) measures scored for the Delay task were not. Importantly, the relations between ToM and performance in the Conflict Task remained significant after controlling for effects due to age and receptive vocabulary. Therefore, individual differences in cool IC, but not hot IC, were related to ToM performance.

**Table 2:** Pearson correlations between behavioral measures.

**Measure**	**1**	**2**	**3**	**4**	**5**	**6**	**7**	**8**	**9**	**10**	**11**	**12**	**13**	**14**
(1) FB Self-attribution	_	0.298**	0.486**	0.213	0.083	–0.072	–0.035	–0.072	–0.061	0.124	0.351	–0.208	0.275**	0.259*
(2) FB Other attribution	0.274**	_	0.851**	0.308**	0.033	0.023	0.067	–0.163	–0.156	–0.164	–0.094	–0.258	0.218*	–0.004
(3) FB Global score	0.494**	0.837**	_	0.303**	–0.051	–0.007	0.070	–0.150	–0.137	–0.146	–0.149	–0.258	–0.190	–0.050
(4) C Correct responses	0.087	0.259*	0.267*	_	0.126	–0.030	0.079	–0.014	0.009	–0.023	0.193	0.095	0.406**	0.307**
(5) D Frequency to touch	0.007	0.003	–0.084	0.072	_	0.195	–0.622**	–0.231*	0.083	–0.321	0.281	0.059	0.177	0.216*
(6) D Frequency to open	–0.009	0.065	0.042	0.048	0.264**	_	–0.224*	–0.553**	0.061	0.224	–0.165	0.016	–0.232*	–0.184
(7) D Latency to touch	–0.041	0.048	0.055	0.023	–0.632**	–0.228*	_	0.470**	0.406**	0.035	0.066	0.211	0.085	0.059
(8) D Latency to open	–0.083	–0.178	–0.159	–0.079	–0.233*	–0.560**	0.445**	_	0.718**	–0.251	0.199	0.069	0.131	0.053
(9) D Time to interruption	–0.035	–0.151	–0.125	–0.036	–0.029	0.059	0.370**	0.709**	_	–0.298	0.082	0.080	0.015	–0.069
(10) DCT-FD	0.156	–0.146	–0.155	0.053	–0.299	0.187	0.013	–0.237	–0.311	_	§	§	–0.097	–0.038
(11) DCT-LSTD	0.340	–0.150	–0.244	0.234	0.270	–0.095	–0.009	0.136	0.080	§	_	§	0.180	–0.045
(12) DCT HSTD	–0.213	–0.324	–0.324	0.092	0.105	0.031	0.237	0.051	0.066	§	§	_	–0.089	–0.302
(13) Age	_	_	_	_	_	_	_	_	_	_	_	_	_	0.597**
(14) Receptive vocabulary	_	_	_	_	_	_	_	_	_	_	_	_	_	_

Bivariate correlations are reported over the diagonal, partial correlations (controlling for age and receptive vocabulary) are reported below the diagonal. *p < 0.05; **p < 0.01. §, False Belief task, N = 98; C, Conflict task, N = 81; D, Delay task, N = 98; DCT, Delay Choice task (FD, Food Delay, N = 20; LSTD, Low-Symbolic Token Delay, N = 20; HSTD, High-Symbolic Token Delay, N = 21); § Correlations were not performed since these were different conditions of the same task (between-subject design).

**Table 2** also reports the relations between the variables measured in each task and, respectively, chronological age and receptive vocabulary.

## Discussion

The present study investigated the relations between performances on a task measuring false belief understanding and three tasks measuring cognitive versus affective aspects of IC in a sample of Italian preschool children.

Considering the range of variations in responses within each task, and the correlation found between ToM and the conflict measure of IC, the performances of 3-and 4-years-old Italian children in the current study can be considered similar to those reported in previous studies conducted with children growing up in Western cultures. Similarly to what has been reported in previous studies, 4-years- old children performed better than 3-years-old children in both the False Belief and the Conflict task, and these measures were positively associated even after controlling for age and receptive vocabulary. A direct comparison with the study by Lecce and Hughes (2010) reporting an advantage of British children over Italian children on ToM tasks is not possible, since their study tested children of a different age group (5- to 6-years-old). The conflict task that we used was slightly different from the classical Day–Night Stroop task employed by Gerstadt et al. (1994). While in the classical Day–Night task the child has to say ‘day’ when shown a card with the moon, in our modified version of the task the child had to say ‘red’ when a blue card was shown. In the first case the child is required to inhibit a symbolic categorization, whereas in the second case the child has to inhibit a perceptual categorization.

As in previous studies (Hala et al., 2003; Carlson et al., 2004b, 2015), the children’s performance in the Delay task did non-correlate with ToM measures. Moreover, we did not find any effect of age on children’s performances in the Delay task. Since only 23% of the 3-year-olds and 46% of the 4-year-olds waited for the entire delay (180 s) without touching the musical box, we can exclude that there was a ceiling effect. However, it cannot be excluded that factors such as fatigue (the Delay task was administered at the end of the session) or experimental setting (kindergarten vs. home) may also have had an impact on children’s performance.

A different developmental pattern emerges from children from non-Western cultures, with studies reporting a lack of association between false belief understanding and composite scores of EF (Lewis et al., 2009). Oh and Lewis (2008) reported that Korean preschoolers tend to be 1 year ahead in executive tasks (including both conflict and delay tasks) in comparison to British children, whereas they are at chance level on false belief measures at 4 years of age. For instance, in the Delay task (Gift delay) the majority of children (72.5%) waited for the entire delay (150 s) to touch a present. As noted by Oh and Lewis (2008), Korean children may be very skilled in delay inhibition probably because their culture places emphasis on patience or because impulsive behavior tends to be punished in Korea. These authors also noted that far Eastern countries such as China, Japan, and Korea have a long tradition of Confucianism. Holding parents, teachers, elders, and authority figures in high respect is an important aspect of Confucianism and this may contribute to young children’s self-control and obedience.

Research on the development of IC has differentiated between cognitive components of self-control, assessed by conflict tasks which demand inhibition and some additional cognitive load (e.g., activate a novel response), and affective components, such as affective decision-making that is measured by delay tasks, which require inhibition of an impulsive response (Carlson and Moses, 2001; Perner et al., 2002; Carlson, 2005; Prencipe and Zelazo, 2005). In the present study, we did not find a correlation between the Conflict task and the Delay task. In our view, one important difference between conflict and delay tasks is that they set different challenges to the child during inhibition. Conflict tasks need a relatively abstract and decontextualized type of inhibition, while delay tasks, which activate emotional and reward factors, require a more affective and context-bound type of inhibition. Our results are in line with Kain and Perner’s (2005) proposal that emotional and reward factors could contribute to the reason why delay tasks are not as strongly associated to ToM tasks as conflict tasks. According to these authors, there is some evidence that the areas of activation in the prefrontal cortex during conflict and ToM tasks may overlap in childhood and not be the same as the neural basis of emotional and reward processing involved during delay tasks (Kain and Perner, 2005). Similarly, we did not find a significant correlation between performance in the Delay Choice task and the Delay task, in agreement with those studies in which only a weak correlation between delay choice and delay maintenance measures (or a lack thereof) was reported for the same population (Schwarz et al., 1983; Duckworth and Kern, 2011; Addessi et al., 2013).

On a theoretical level, alternative reasons have been proposed to explain the developmental link between self-control and ToM (see Perner and Lang, 1999 for a review). With respect to expression views (Hughes and Russell, 1993), good performances on standard ToM tasks demand some level of executive ability to inhibit the true state of affairs and reflect on mental states. With respect to emergence views, there is a functional and ontogenetic relationship between EF and ToM, but several proposals have been put forward for the developmental direction. According to Perner and Lang (1999), an understanding of mental states as causally effective representations is necessary for the development of self-monitoring and IC. On the other hand, Russell (1996) and Carlson and Moses (2001) hypothesized that a certain level of executive control could be essential for the acquisition of mental state concepts. There is not yet definitive proof backing the expression or the emergence view (Henning et al., 2011; Devine and Hughes, 2014) and testing the different theoretical accounts of the relation between IC and ToM is clearly beyond the scope of our work.

Our study, even with its limitations (including not using a wide battery of EF and ToM tasks, and the concurrent measurements that precludes clear causal statements) supports the results of those studies that found a significant relation between the ability to understand false beliefs and the conflict (but not the delay) measures of IC. Moreover, in the present study, delay and conflict measures were not associated, suggesting that these tasks measure different components of IC. The dissociation between the Conflict and the Delay task may indicate that monitoring and regulating a hot process (not touching an available and highly attractive stimulus) may involve different abilities than monitoring and regulating a cool process (as flexible categorization), and that only the latter component of IC is developmentally linked to false belief understanding. Also performance in the Delay Choice task and in the Delay task did show a lack of correlation, probably because these two tasks tackle different aspects of delay of gratification ability. Whereas in the Delay Choice task the initial choice cannot be reconsidered during the delay, in the Delay task the subject can modify her choice at any time.

The lack of correlation between ToM and “hot” IC, observed in previous studies and replicated in the present study, has important implications on the alleged link between delay of gratification and so-called “mental time travel” (MTT). MTT is defined as the ability to mentally project oneself in some future situation (Atance and Meltzoff, 2005; Suddendorf and Corballis, 2007), and it is increasingly conceptualized as continuous and complementary with the ability to remember episodes on one’s past (episodic memory; Busby and Suddendorf, 2005; Addis et al., 2007). MTT has often been suggested as a key element for planning (Atance and Meltzoff, 2006) and delayed gratification: as Atance (2008, p. 297) argued “were an organism not able to conceptualize a time other than the present, then delaying would make little sense.” In turn, MTT is typically described as a complex faculty that relies on a variety of cognitive processes, including ToM, IC, and working memory (Suddendorf and Corballis, 2007). According to this view, (i) ToM and “hot” IC should positively correlate, through the mediation of MTT, and (ii) subjects that are unable to mentally project themselves in future situations should demonstrate substantial impairment in delay tolerance. The latter hypothesis is contradicted by recent data on amnesic individuals with hippocampal damage and associated impairments in episodic memory and future imagining (Kwan et al., 2012, 2013): in spite of their impaired MTT abilities, these subjects exhibited the same delay discounting behavior observed in demographically matched controls, thus suggesting that MTT is not a necessary condition for delay tolerance. As for the idea that ToM may facilitate “hot” IC, this is at odds with previous developmental evidence (Carlson and Moses, 2001; Carlson et al., 2002, 2015; Hala et al., 2003; Kain and Perner, 2005; Devine and Hughes, 2014), as well as with the present findings.

The impact of our results on the MTT debate is moderated by the fact that we did not check for MTT abilities specifically, so we cannot be sure that the age-related improvement in ToM observed in our sample also resulted in a similar improvement in MTT skills (although this would be consistent with the relationship between ToM and MTT hypothesized by most MTT scholars). Nonetheless, in the present study ToM did not correlate with performance in either the Delay task or the Delay Choice task: the latter finding, in particular, suggests a lack of role for ToM in “hot” IC. However, as for the Delay Choice task, it cannot be excluded that the small sample size might have played a role, and future studies should evaluate whether performance in ToM tasks and in the Delay Choice task correlates in larger samples. More generally, this discussion shows that (i) the connection between ToM and delayed gratification hypothesized by proponents of MTT as a key aspect of future-oriented self-control (Suddendorf and Corballis, 2007; Atance, 2008) is far from being proven, and thus (ii) a more comprehensive examination of the relationship between ToM and different aspects of IC is urgently needed.

## Conclusion

Although we did not test children with a full battery of EF and ToM tasks, our results are stimulating and broadly in line with previous findings. Future research on the relationship between ToM and IC should include several conflict and delay measures and test children in different cultures in order to better understand the role of cognitive vs. affective components of self-control and their specific relation to ToM development. A better understanding of the interdependence between ToM and IC may also come from a thorough investigation of their neural basis and evolutionary precursors, via comparative studies.

## Author Contributions

Conceived and designed the experiments: FB, EA, FP. Performed the experiments: VF, GP, VM, BP. Analyzed the data: FB, EA, GP. Wrote the paper: FB, EA, GP, FP.

## Conflict of Interest Statement

The authors declare that the research was conducted in the absence of any commercial or financial relationships that could be construed as a potential conflict of interest.
